# An Assessment of the Public's Perceptions of Radiation Exposure and Risk Associated With Dental Radiographs: A Cross-Sectional Study

**DOI:** 10.7759/cureus.47879

**Published:** 2023-10-28

**Authors:** Lyan Y Qari, Najwa J Homsi, Tamara M AlMadani, Duaa M Jamal, Fatma F Badr

**Affiliations:** 1 Faculty of Dentistry, King Abdulaziz University, Jeddah, SAU

**Keywords:** dental x-ray, equivalency, background radiation, public knowledge, radiation risk

## Abstract

Background: Ionizing radiation exposure is an ever-present part of the dental diagnostic process. A public concern often exists due to the misunderstanding of the stochastic effects of dental X-rays. This information can be difficult to explain to the patient since many patients are apprehensive about the subject matter.

Objective: This article aims to assess the public's knowledge of radiation exposure and estimate the general concern or apprehension about these diagnostic imaging modalities in an effort to understand and therefore ensure patient reassurance during treatment.

Method: A questionnaire was conducted asking adults between the ages of 18 to 74 in Jeddah, Saudi Arabia questions pertaining to radiation risk.

Results: There were 105 respondents; 21.9% showed concerns toward dental imaging, while 20% were skeptical. approximately 74% of respondents believed there was a limit to the amount of radiation exposure a patient could receive for diagnostic purposes, while only eight percent correctly identified that there was no set limit. Only 21.9% knew that a breastfeeding mother could have dental X-rays if need be; 33.3% understood that ionizing radiation from an intra-oral dental X-ray caused less exposure than natural background radiation from a return flight from Jeddah to Dammam.

Conclusions: Patients are not aware of ionizing radiation exposure equivalencies between different imaging modalities. A more effective approach to convey exposure risk would be relating the radiation doses to natural background radiation as comparators.

## Introduction

Radiation exposure associated with dental examination is a routine aspect of all dental procedures; however, misinformation and misunderstanding regarding risks associated with ionizing radiation (IR) have created a general public concern [1]. This stigma could result in the overall avoidance of these diagnostic procedures and refusal of treatment [2],^ ^which could ultimately lead to compromised management. IR is an ever-present environmental stress [3]. It can arise from radiographic imaging and is a constant and natural part of our everyday accumulated dose of background radiation [4]. However, in accordance with the linear no-threshold model, X-rays and computed tomography imaging techniques employing IR carry a stochastic lifetime risk of developing malignancy [4-6]. This conflict of interest favors the use of these imaging modalities since the diagnostic and treatment benefits far outweigh its risks [6].

Members of the general public may find it difficult to fully comprehend the risks associated with IR and the protective radiation safety measures mandated by law [7]. According to the most recent online scientific database published in the field of radiation protection and safety for healthcare providers in Saudi Arabia and the data published in the KSA-SFDA report in 2022 [8], the update emphasizes the importance of physician or radiologist disclosure and complete transparency of the possible associated side effects, hazards, and pre- and post-procedural instructions with the patient prior to any radiation exposure. In an effort to determine an effective disclosure protocol that effectively communicates the benefits and risks of IR to patients, it is first necessary to evaluate the current level of knowledge as well as study the perceptions that exist about radiation in the community. The first objective of this study is to assess the public's knowledge of radiation exposure. The second objective is to estimate the general fear or apprehension about dental imaging modalities. The third objective is to determine the attitude of participants toward improving the quality and reducing the exposure during dental imaging. The final objective is to explore any significant associations between demographic factors and knowledge or fear or attitude.

## Materials and methods

Participants

This cross-sectional study received ethical approval from the Research Ethics Committee of King Abdulaziz University Faculty of Dentistry, Jeddah Saudi Arabia. Following approval, 105 respondents were recruited using snowball sampling and information was spread through social media. The bilingual survey was distributed online over a period of three months between January and March of 2022.

The survey was designed to assess public awareness of radiation exposure from dental X-rays relative to natural background radiation. In addition to asking some questions that help address everyday common misconceptions and comparing high- and low-dose modalities, the survey was then analyzed and compiled using Google Forms.

Respondents were required to be over 18 years of age, understand the questionnaire in either English or Arabic, and be members of the general public. The survey did not include current patients, and there was no requirement to have or have had any previous imaging.

Questionnaire 

Four sections were included in the survey. In the first section, demographic information was collected (gender, age range, and occupation) in order to characterize the sample. Participants were also asked if they were aware of certain imagining modalities on a yes or no basis (Table 1). 

**Table 1:** Descriptive analysis of demographic variables (n=105)

Variable	Group	Frequency	Percentage
Gender	Male	29	27.6%
	Female	76	72.4%
Age Group	18-25	49	46.7%
	25-34	32	30.5%
	35-44	5	4.8%
	45-54	9	8.6%
	55-64	8	7.6%
	65-74	2	1.9%
Occupation	Business/IT	25	23.8
	Housewives/Student/Retired	53	50.5%
	Science/Health care	14	13.3
	Others	13	12.4
Magnetic Resonance Imaging Awareness	No	87	82.9%
	Yes	18	17.1%
Cephalometric radiograph Awareness	No	100	95.2%
	Yes	5	4.8%
Panoramic radiograph Awareness	No	54	51.4%
	Yes	51	48.6%
Cone Beam Computed Tomography Awareness	No	96	91.4%
	Yes	9	8.6%
Intra-oral dental radiograph Awareness	No	45	42.9%
	Yes	60	57.1%

The second section aimed to evaluate a “knowledge score”; eight questions related to diagnostic imaging modality awareness were asked. These questions aimed to evaluate knowledge of basic aspects of radiation awareness. The options given to answer the first six questions in this section were “strongly agree”, “agree”, “strongly disagree”, and “disagree”. During analysis, respondents who either agreed or strongly agreed with the statement were combined. Similarly, those who disagreed or strongly disagreed were also combined. For those in between, “neither agree nor disagree” was a response option. The seventh question in this section asked respondents to assign five different imagining modalities a radiation dose that they thought corresponded to each’s level of natural background radiation. Finally, the eighth question asked respondents to rank different forms of radiation exposure according to what they believed exposed them to more IR, on a scale of 1 (lowest) to 5 (Table 2). A knowledge score was created for each participant by calculating the mean of all nine knowledge questions answered, between 0 (if all answers were incorrect) and 1 (if all answers were correct). The maximum knowledge score was 0.63 out of 1. 

**Table 2:** Outcome 1: Knowledge questions (and correct answers) “Knowledge” score CBCT: Cone beam computed tomography

Knowledge Questions “Knowledge Score”	Strongly Disagree		Disagree			Neither agree nor disagree			Agree		Strongly Agree			Frequency of correct responses		
A person who has had an X-ray is radioactive for 24 hours (no)	15 (14.3%)		33 (31.4%)			37 (35.2%)			14 (13.3%)		6 (5.7%)			48 (45.7%)		
A breastfeeding mother can have a dental X-ray (Agree, strongly agree)	24 (22.9%)		32 (30.5%)			26 (24.8%)			19 (18.1%)		4 (3.8%)			23 (21.9%)		
There is a limit to the amount of X-ray radiation a patient can have in a year for medical evaluation (Disagree, strongly disagree)	2 (1.9%)		7 (6.7%)			18 (17.1%)			44 (41.9%)		34 (32.4%)			9 (8.6%)		
A child is 𝗺𝗼𝗿𝗲 at risk from X-ray radiation than an adult (Agree, strongly agree)	3 (2.9%)		11 (10.5%)			32 (30.5%)			38 (36.2%)		21 (20%)			59 (56.2%)		
A pregnant woman 𝗰𝗮𝗻𝗻𝗼𝘁 have a dental X-ray (she can under certain restrictions)	7 (6.7%)		20 (19%)			25 (23.8%)			33 (31.4%)		20 (19%)			52 (49.5%)		
A pregnant woman should avoid a person who had an X-ray earlier that day (Disagree, strongly disagree)	22 (21%)		34 (32.4%)			33 (31.4%)			13 (12.4%)		3 (2.9%)			56 (53.4%)		
A CBCT (3D imaging) and intra-oral dental X-ray give the same amount of radiation (Disagree, strongly disagree)	8 (7.6%)		23 (21.9%)			47 (44.8%)			19 (18.1%)		8 (7.6%)			31 (29.5%)		
Select the equivalent amount of exposure you believe to correspond to each type of X-ray:		Head CT			Knee Radiograph			Full mouth intra-oral x rays				Set of intra-oral bitewing radiographs			
6 months (Head CT)		14 (13.3%)			6 (5.7%)			6 (5.7%)				6 (5.7%)			
1 hour (Knee Radiograph)		26 (24.8%)			28 (26.7%)			35 (33.3%)				35 (33.3%)			
2-4 days (Full mouth intra-oral X-rays)		20 (19%)			15 (14.3%)			20 (19%)				16 (15.2%)			
Half a day (Set of intra-oral bitewing radiographs)		17 (16.2%)			29 (27.6%)			20 (19%)				20 (19%)			
I don’t know		28 (26.7%)			27 (25.7%)			24 (22.9%)				35 (33.3%)			
Please rank the following in order of radiation exposure, where 1 is the lowest radiation exposure	1			2			3			4			5			
Intraoral dental X-ray (0.001-0.002 mSv)	33.3%			21.9%			35.2%			7.6%			1.9%			
Return flight from Jeddah to Dammam (0.03-0.04 mSv)	51.4%			27.6%			11.4%			5.7%			3.8%			
Chest X-ray (0.1 mSv)	20%			21.9%			25.7%			16.2%			16.2%			
Annual average dose from radon in Saudi Arabia (0.5-1 mSv)	29.5%			26.7%			25.7%			11.4%			6.7%			
Chest CT (6 mSv)	23.8%			8.6%			26.7%			16.2%			24.8%			

The third section focused on asking questions that would evaluate participant’s fear of radiation. Asking participants if they thought dental X-rays were dangerous, if they had any concerns about imaging, and if they would refuse taking a dental X-ray for their child out of fear of radiation (Table 3). A fear score was then extracted for each participant from all fear/ apprehension questions answered, between 0 (if all answers were incorrect) and 1 (if all answers were correct). The maximum fear score was 1 out of 1.

**Table 3:** Outcome 2: Fear questions (and correct answers) “Fear” score

Fear Questions “Fear Score”	Yes	No	Maybe	Not Applicable
Do you consider dental X-rays dangerous? (yes)	18 (17.1%)	43 (41%)	44 (41.9%)	
Do you have any worries or concerns about having a dental X-ray? (yes)	23 (21.9%)	61 (58.1%)	21 (20%)	
Would you refuse taking a radiograph for your child in a dental clinic out of fear of radiation? (yes)	12 (11.4%)	50 (47.6%)	37 (35.2%)	6 (5.7%)

The final section assessed attitude, asking questions to see if respondents were willing to go out of their way for better image quality and lower dose imaging. These results were then used to calculate a “positive attitude score”. (yes: positive, no: negative). The maximum attitude score was 1 out of 1.

## Results

A questionnaire of 105 respondents was administered and collected. On a scale from 0 to 1, the mean knowledge score was 0.27 +/- 0.13 standard deviation (SD). The mean fear/apprehension score was 0.17 +/- 0.24 SD. The mean attitude score was 0.46 +/- 0.43 SD.

The majority of participants (72.4%) were females, nearly half (46.7%) were between 18 and 25. Thirty percent (30.5%) were between 25 and 34 years old, and half of our participants were housewives, students, or retired. 23.8% of them were working in Business, education, finance, or IT (Table 1). The Shapiro-Wilk test of normality was performed to test the distribution of data. The data violates normality assumption; therefore, the Mann-Whitney U test was conducted to test the difference in the mean knowledge score between males and females. Also, two Kruskal-Wallis tests were performed to examine the difference in the knowledge score between the age groups and between the occupation groups. There is no significant difference in knowledge score between genders, age groups, or occupation groups p>.05.

The sample was most aware of intra-oral dental radiographs (57.1%), followed by panoramic radiographs (48.6%), followed by MRI (17.1%), followed by cone beam computed tomography (CBCT) (8.6%), and least aware of cephalometric radiographs (4.8%).

The second section asked eight questions. The first question starts by asking if patients receiving radiotherapy are radioactive for 24 hours. To which most respondents chose to neither disagree nor agree (35.2%), while the correct response (no) received a combined response of 48 (45.7%). The second question showed that 21.9% of people incorrectly believe that a breastfeeding mother cannot have a dental X-ray. Similarly, most participants (74.3%) suppose that there is a limit to the amount of radiation a patient can receive for medical evaluation, while only 8.6% correctly identified that there is not. Respondents who correctly identified that children are more at risk from radiation were at 56.2%, while wrong answers cumulatively added up to 13.4%. Similarly, many respondents correctly identified that a pregnant woman could have a dental X-ray under certain restrictions if need be at 49.5%; 53.4% of patients also correctly chose that there is no need for a pregnant woman to avoid patients after X-ray acquisition, whilst 15.4% believed they should, and 31.4% were unsure. 

With regard to patient’s knowledge of the technology utilized, 70.5% were unaware that the use of 3D technology in acquisition exposed them to more radiation than the average 2D X-ray image, whilst only 29.5% came to that conclusion. The seventh question of section 1 revealed most respondents incorrectly identified full mouth intra-oral X-rays and a set of bitewings as causing the least amount of radiation exposure, and only 13.3% of respondents assumed that a head CT was equivalent to six months of background radiation; 26.7% correctly identified the knee radiograph as causing the least radiation exposure, and 19% correctly identified the set of intra-oral bitewings, and intra-oral X-rays in their relative positions. An overwhelming 22.9-26.7% claimed they did not know the relative positions of each modality, with “I don’t know” being the highest response for head CT imaging at 26.7%.

The eighth question asked the respondents to rank the radiation exposure of each imaging modality to their knowledge. Most respondents seemed to think that a return flight from Jeddah to Dammam caused the least radiation exposure, when in fact an intra-oral dental X-ray actually exposes one to less radiation; 27.6% of respondents then correctly chose the return flight from Jeddah to Dammam as the second lowest radiation exposure dose. For third place, most respondents believed that the intra-oral dental X-ray was ascribed to that position when really the chest X-ray is third in rank. In the fourth position, only 11.4% chose the annual average dose from radon in the home and workplace correctly, while most respondents believed a chest CT or X-ray would expose an individual to a higher radiation dose, with 16.2% of respondents picking either. Lastly, 24.8% of respondents correctly assumed that the chest CT would lead to the highest exposure between the given choices, with the least picked for the rank being the intra-oral dental X-rays, followed by the return flight from Jeddah to Dammam.

The second section of the survey showed most participants (41.9%) were unsure if dental radiographs were dangerous. However, 58.1% claimed they had no worries or concerns about having imaging done. This demonstrates that the majority of the study sample is not apprehensive about dental imaging. Finally, 11.4 % of respondents would refuse radiographic imaging for their children out of fear of radiation exposure, while 47.6% had no such concern. 

Section 3 showed almost half of the participants (44.8-46.7%) were willing to drive an extra hour for better quality and lower dose imaging (Table 4). Males (65.5%) showed a more positive attitude than females (56.6%), and more fearlessness (65.5%) than females (60.5%), although the difference was statistically insignificant. 

**Table 4:** Outcome 3: Positive attitude questions (and correct answers) “Positive Attitude” score

Positive attitude Questions “Positive Attitude” score	Yes	No	Maybe
If a new dental X-ray device were available that used significantly less radiation, would you be prepared to drive an extra hour to a hospital with this technology (yes)	49 (46.7%)	28 (26.7%)	28 (26.7%)
If a new dental X-ray device were available that produced better quality imaging, would you be prepared to drive an extra hour to a hospital with this technology (yes)	47 (44.8%)	22 (21%)	36 (34.3%)

**Figure 1:**
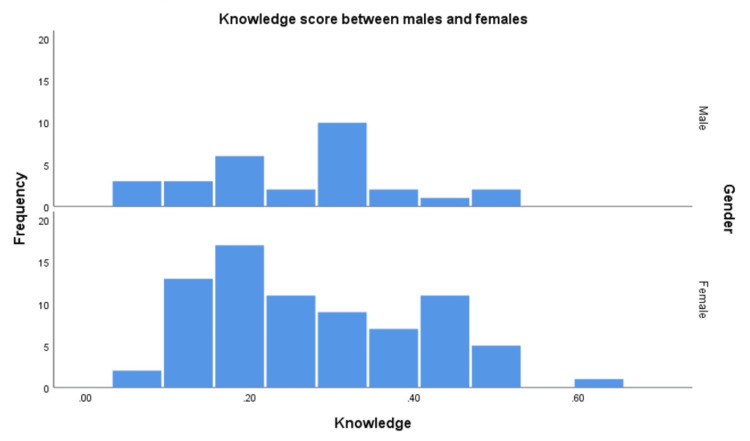
Frequency of knowledge scores between males and females

**Figure 2:**
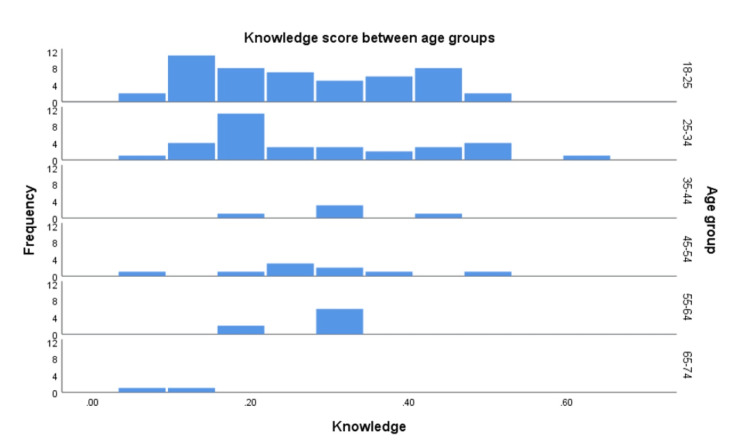
Frequency of knowledge scores between age groups

A linear regression model was applied (Table 5) to check the effect of predictors on knowledge outcome, this model showed a significant fit to data f(96,9) = 33.133, p<.000. The R square is 87.0%, according to our model, the males had nearly one-sixth of the knowledge compared to females β= .146 (Figure 1). The knowledge score in older age groups seems to be lower than the youngest 18-25 group; for example, participants aged 25-34 years had lower knowledge by 0.1 points compared to the 18-25 group, p<.05 (Figure 2). For occupation comparison, the business/IT group was chosen as the reference comparison group, housewives, students, and retired individuals had half the knowledge of the business/IT group. 

**Table 5:** Linear regression odds ratios for factors associated with knowledge (n=105). Model adjusted for gender, age, and occupation. p-values: * p ≤ 0.05, ** p ≤ 0.01, *** p ≤0.001.

	B	t	p-value	95.0% Confidence Interval for B	
				Lower Bound	Upper Bound
Gender	.146	4.092	.000	.075	.217
Age group					
25-34	.100	2.926	.004	.032	.168
35-44	.269	3.842	.000	.130	.407
45-54	.154	2.899	.005	.049	.259
55-64	.176	3.000	.003	.060	.292
65-74	-.004	-.038-	.970	-.234-	.226
Occupation					
Housewives/Student/Retired	.098	2.576	.012	.023	.174
Science/Health care	.091	1.718	.089	-.014-	.196
Others	.073	1.434	.155	-.028-	.175

**Figure 3:**
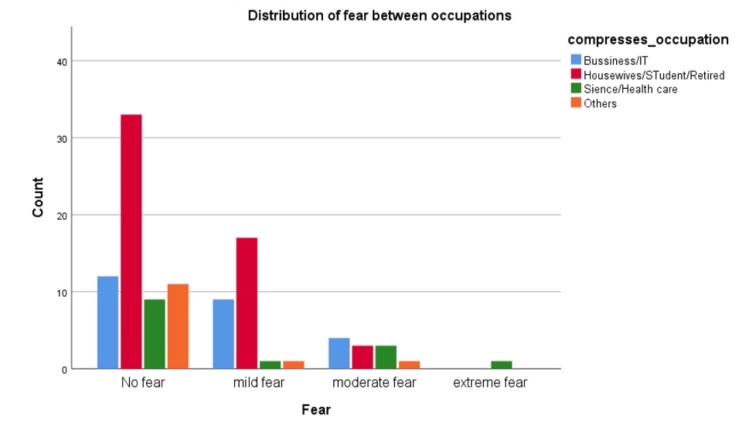
Distribution of fear between occupations

A logistic regression model was applied (Table 6) to check the effect of predictors on fear outcome, the R square is 19.4%, and the only significant predictors are the age group 25-34 years as their odds ratio is 0.161 compared to the younger group 18-25 years old; this means getting older decreases the probability of being afraid from procedures by 0.84 from the younger group. Moreover, Housewives/Students/Retired had 5.56 times probability of being afraid compared to the business/IT group P<.05 (Figure 3).

**Table 6:** Logistic regression odds ratios for factors associated with fear (n=105). Model adjusted for gender, age, and occupation. p-values: * p ≤ 0.05, ** p ≤ 0.01, *** p ≤0.001.

	B	S.E.	Wald	p-value	Exp(B)	95% C.I.for EXP(B)	
						Lower	Upper
Gender(males)	-.775	.581	1.782	.182	.461	.148	1.438
Age group			8.489	.131			
25-34	-1.826	.738	6.118	.013	.161	.038	.685
35-44	-1.100	.715	2.363	.124	.333	.082	1.353
45-54	-1.250	1.229	1.034	.309	.286	.026	3.188
55-64	-.366	1.013	.130	.718	.693	.095	5.053
65-74	-1.634	1.092	2.237	.135	.195	.023	1.660
Occupation			4.228	.238			
Housewives/Student/Retired	1.715	.860	3.976	.046	5.557	1.030	29.993
Healthcare/Science	1.116	.729	2.342	.126	3.054	.731	12.759
Other	.811	.895	.822	.365	2.251	.390	13.005

A logistic regression model was applied to check the effect of predictors on attitude outcome (Table 7). No significant predictors except the age group of 55-64 years showed a p value= 0.05 which can be considered significant, yet the confidence interval was very broad which cannot be accepted.

**Table 7:** Logistic regression odds ratios for factors associated with attitude (n=105). Model adjusted for gender, age, and occupation. p-values: * p ≤ 0.05, ** p ≤ 0.01, *** p ≤0.001.

	B	S.E.	Wald	p-value	Exp(B)	95% C.I.for EXP(B)	
						Lower	Upper
Gender(males)	.120	.578	.043	.836	1.127	.363	3.499
Age group			7.075	.215			
25-34	.772	.492	2.460	.117	2.163	.825	5.672
35-44	1.925	1.256	2.350	.125	6.855	.585	80.324
45-54	1.135	.849	1.787	.181	3.113	.589	16.447
55-64	2.367	1.208	3.841	.050	10.669	1.000	113.854
65-74	-.430-	1.552	.077	.782	.651	.031	13.617
Occupation			5.586	.134			
Business/IT	-.756-	.610	1.537	.215	.469	.142	1.552
Housewives/Student/Retired	.310	.320	.940	.332	1.363	.729	2.550
Healthcare/Science	-1.177-	.690	2.915	.088	.308	.080	1.190

## Discussion

In recent years, remarkable advancements in diagnostic and therapeutic radiology have been integrated into the field of dental science. Advancements in the field have improved the effectiveness, decreased the complications, and expanded the implications of radiation therapy [9]. With great strides in therapy came increased sources of radiation. Natural radioactivity alone approximates 82% of the amount of radiation absorbed by humans daily [10]. These exposures come from natural sources, such as cosmic radiation, terrestrial radiation, and exposure through inhalation [10]. The fraction of radiation that does manifest through different forms of medical diagnostic procedures is what may bring out people’s concern. Some of these concerns include genetic effects and neoplasms, such as primary bone tumors, leukemia, or thyroid carcinoma [11]. 

Healthcare workers are relied upon by patients to provide up-to-date information on radiation from medical exposures. This information must be communicated in accordance with new legislation [8]. Overall, our results demonstrate that respondents only have a general understanding of IR. For example, standard precautions regarding pregnancy have given the public some idea of the risks of radiation. Table 2 shows that just over 50% of our respondents understand that a pregnant woman may take X-rays under certain restrictions; however, 53.4% of respondents believe that a breastfeeding mother should not. Those surveyed seem to not understand that it is the healthcare provider or in our case the dentist’s decision to evaluate the risk and benefit, and if the benefits outweigh the risks, safety measures are undertaken to minimize any unwanted exposure and reduce the radiation that could penetrate the body.

Our questionnaire started with demographic data (Table 1). Then participants were given statements to answer depending on what they already knew (Table 2). Starting with the first statement, less than half of the participants 45.7% answered correctly that a person who has had an X-ray cannot be radioactive for 24 hours. Where in a previous study, 72% knew the correct answer to this question [12]. Only 21.9% agreed that a breastfeeding mother could have a dental X-ray, while in another study 57% agreed with this statement [12]. This demonstrates that more than 50% of respondents think that radiation can affect breast milk and that it is dangerous. There is no difference between a breastfeeding mother and another healthy individual. There are no studies that prove any danger related to dental X-rays during this time. Even a pregnant woman can wear a lead apron ensuring protection of the fetus when used with all the other radiation protection measures [13]. But as shown in these results, the public still does not believe this with 50% refusing to let a pregnant woman have a dental X-ray and 23.8% not actually knowing what the right thing to do is.

The pregnancy topic seems to be confusing for the public when it comes to dental X-rays or anything related to radiation and radiology. In another question, 31.4% neither agreed nor disagreed with a pregnant woman having to avoid a person who had an X-ray that day. A person does not become radioactive after a few dental X-ray images. Thus, it is fine for a pregnant woman to meet someone after having an X-ray that day. Unlike another study, 73% of the participants understood that it is no problem for a pregnant woman to be near someone who just had a dental X-ray [12]. 

When it comes to the amount of radiation exposure, 8.6% were correct about the patient not having a yearly limit for medical evaluation. When asked if the amount of radiation from a CBCT and an intra-oral dental X-ray are the same, less than 30% answered correctly that there is a difference, this information suggests significant misconceptions in terms of which modalities give off higher levels of IR. Lastly, more than half of respondents knew that a child is more at risk of developing cancer after radiation exposure [14]. 

As shown in Table 2, respondents were asked about exposure from different imaging modalities and the matching natural background radiation equivalent. For example, a set of full mouth intraoral X-rays corresponds to about 2-4 days of natural background radiation. According to our results, all modalities were only correctly matched less than 30% of the time. A previous study shows that most participants also think that radiation from artificial sources shows a higher risk than that of natural background radiation [12].

The last part of section 2 asked respondents to rank everyday sources of radiation according to their level of radiation exposure. 33.3% said that intra-oral dental X-rays are the lowest among all the other sources which they are correct about. This is close to the result of a previous study that showed 33% answered correctly as well [12]. 

Regarding the fear questions, 41.9% considered dental X-rays to be dangerous, and 11.4% would not let their child take a dental X-ray out of fear of radiation; however, 58.1% have no worries regarding dental X-rays. If approximately one in every ten patients would refuse to let their child have a dental X-ray, this implies that there is a concept that needs to be rectified by increasing public education on this matter.

From all of our collected responses to all these different questions, we can assume that to this day people are still confused regarding precautionary measures of IR. Public education can start from dental clinics, by listening to the patient’s thoughts and concerns and ensuring that they come out of the clinic with the correct answers to all their worries and concerns.

As for some limitations to the study, snowball sampling was used to collect responses, which poses a limitation to the study. Snowball sampling is the process of referring a survey to other participants who may be interested in the topic. As effective as snowball sampling is when it comes to sharing online surveys, it is not ideal and can result in biased results. The sample size of the study also could have been larger, incorporating more people into the study could lead to more accurate results. Also, some of the questions may have been difficult to translate and therefore answer by the public. However, providing a bilingual questionnaire was done to minimize difficult interpretation of the questions.

## Conclusions

The deficiency in public awareness regarding radiation exposure, coupled with prevalent apprehension and concern associated with dental radiography, underscores a notable knowledge gap concerning the actual extent of radiation to which dental patients are subjected. In order to augment awareness and mitigate apprehension, it is imperative for dental healthcare professionals to enhance their proficiency in effective patient communication, thereby facilitating a transition from "X-ray anxiety" to a state of heightened awareness regarding dental imaging.

In addition to the key findings presented, it is noteworthy that our analysis revealed significant disparities in knowledge and fear levels based on gender and occupation. Males exhibited a notably lower level of knowledge compared to females. Furthermore, our research identified a substantial difference in fear levels among various occupational groups, with housewives, students, and retired individuals demonstrating significantly higher fear levels in contrast to business and IT professionals. These statistically significant results emphasize the necessity of tailored approaches in patient education and communication strategies to address the unique needs and concerns of different demographic and occupational groups, ultimately contributing to more effective dental healthcare practices and improved patient experiences.

## Appendices

An Assessment of the Public's Perceptions of Radiation Exposure and Risk Associated with Dental Radiographs                                                              

تقییم وجھة نظر العامة عن التعرض للإشعاع والمخاطر المرتبطة بتصوير الأشعة للأسنان

1. Gender / الجنس:

O Male / ذكر

O Female / انثى

2. Age group / الفئة العمرية:

O 18-24 / ١٨- ٢٤

O 25-34 / ٢٥- ٣٤

O 35-44 / ٣٥- ٤٤

O 45-54 / ٤٥- ٥٤

O 55-64 / ٥٥- ٦٤

O 65-74 / ٦٥- ٧٤

O 75-84 / ٧٥- ٨٤

O 85+ / ٨٥+

3. Occupation / القطاع الوظيفي:

O Administration / الإدارة

O Business / إدارة أعمال

O Education / التعليم

O Engineering / الهندسة

O Finance / المالیة

O Healthcare / القطاع الصحي

O Homemaker / رب/ة منزل

O IT / تقنية المعلومات

O Law / محاماة

O Media / اعلام

O Public service / خدمة عامة

O Science / القطاع العلمي

O Student / طالب/ة

O Tradesperson / التجارة

O Retired / متقاعد/ة

O Other / غیرھا

4. Have you ever had any of the following exams? (tick all that apply)                                                                                                                        

ھل سبق لك إجراء أي من الاختبارات التالية؟  (اختر كل ما ینطبق)

O Intra-oral dental radiograph / الاشعة السینیة للأسنان

O Panoramic radiograph / الاشعة السینیة البانورامیة

O Cone Beam Computed Tomography / الاشعة المقطعية المخروطية

O Cephalometric radiograph / أشعة الرأس السيفالومترية

O Magnetic Resonance Imaging / التصویر بالرنین المغناطيسي

Please answer the following questions with your own ideas and perceptions of radiation exposure                                                                                                      

الرجاء الإجابة على الأسئلة التالية بأفكارك ووجهة نظرك الخاصة عن التعرض للإشعاع

5. A person who has had an X-ray is radioactive for 24 hours?                                                                                       

الشخص الذي تعرض للأشعة السینیة یكون مشعاً (مصدر للأشعة) لمدة ٢٤ ساعة

O Strongly agree / موافق بشدة

O Agree / موافق

O Neither agree nor disagree / محاید

O Disagree / غیر موافق

O Strongly Disagree / غیر موافق بشدة

6. Do you consider dental X-rays dangerous?   

ھل ترى أن الأشعة السینیة للأسنان خطيرة؟

O Yes / نعم

O No / لا

O Maybe / ربما

7. Do you have any worries or concerns about having a dental X-ray?                                                                            

ھل لدیك أي مخاوف بشأن إجراء أشعة سینیة للأسنان؟

O Yes / نعم

O No / لا

O Maybe / ربما

8. A breastfeeding mother can have a dental X-ray?                                                                                                                                                     

بإمكان الأم المرضعة إجراء أشعة سینیة للأسنان؟

O Strongly agree / موافق بشدة

O Agree / موافق

O Neither agree nor disagree / محاید

O Disagree / غیر موافق

O Strongly disagree / غیر موافق بشدة

9. Would you refuse taking a radiograph for your child in a dental clinic out of fear of radiation?

اذا كان لديك اطفال هل ترفض أخذ صورة أشعة لطفلك في عيادة الأسنان خوفًا من التعرض للإشعاع؟

O Yes / نعم

O No / لا

O Maybe / ربما

O Not applicable / لا ينطبق

10. There is a limit to the amount of X-ray radiation a patient can have in a year for medical evaluation?                                                                                                                                         

هناك حد لمقدار الأشعة السینیة التي یمكن أن یحصل علیھا المریض في السنة للتقییم الطبي؟

O Strongly agree / موافق بشدة

O Agree / موافق

O Neither agree nor disagree / محاید

O Disagree / غیر موافق

O Strongly disagree / غیر موافق بشدة

11. A child is 𝗺𝗼𝗿𝗲 at risk from X-ray radiation than an adult?                                                                                      

الطفل معرض لخطر الأشعة السینیة أكثر من الكبار؟

O Strongly agree / موافق بشدة

O Agree / موافق

O Neither agree nor disagree / محاید

O Disagree / غیر موافق

O Strongly disagree / غیر موافق بشدة

12. A pregnant woman 𝗰𝗮𝗻𝗻𝗼𝘁 have a dental X-ray?                                                                                                            

لا یمكن للحامل إجراء أشعة سینیة للأسنان؟

O Strongly agree / موافق بشدة

O Agree / موافق

O Neither agree nor disagree / محای

O Disagree / غیر موافق

O Strongly disagree / غیر موافق بشدة

13. A pregnant woman should avoid a person who had an X-ray earlier that day?                                                             

یجب على الحامل أن تتجنب الشخص الذي تعرض للأشعة السینیة لمدة یوم؟

O Strongly agree / موافق بشدة

O Agree / موافق

O Neither agree nor disagree / محاید

O Disagree / غیر موافق

O Strongly disagree / غیر موافق بشدة

14. A CBCT (3D imaging) and intra-oral dental X-ray give the same amount of radiation?                                                             

ینتج التصوير المقطعي والأشعة السینیة للأسنان (داخل الفم) نفس الكمیة من الإشعاع؟

O Strongly agree / موافق بشدة

O Agree / موافق

O Neither agree nor disagree / محاید

O Disagree / غیر موافق

O Strongly disagree / غیر موافق بشدة

يتم التعبير عن جرعة الاشعة الناتجة من الفحوصات الطبية، بمعادلتها بمقدار ما يتعرض له الانسان من الاشعة الطبيعية على سطح الارض. على سبيل المثال، تعادل الأشعة السينية للعمود الفقري كمية الإشعاع الطبيعي الذي نتعرض له خلال مدة ٤ أشهر من الحياة 

Radiation dose received from medical imaging exams is often given in terms of the equivalent amount of radiation exposure from the earth, called natural background radiation. For Example, an x ray of the spine is equivalent to natural background radiation for 4 months.

15. Select the equivalent amount of exposure you believe to correspond to each type of x-ray:                                

:حدد مقدار التعرض لإشعاع الخلفية الطبيعي الذي تعتقد أنه يتوافق مع كل نوع من أنواع الأشعة السينية           

**Table 8:** Question 15

	One hour/ساعة واحدة	نص يوم/ Half a day	Two-four days/٢-٤ ايام	6 months/٦ شهور	I don't know/لا اعلم
Head CT/الأشعة المقطعية للرأس					
Knee radiograph/أشعة الركبة					
Full mouth intraoral x-rays/أشعة داخل الفم لجميع الأسنان العلوية والسفلية					
Set of intraoral bitewing radiographs/أشعة داخل الفم لجميع الأسنان فقط ،صور مستطيلة					

16. Please rank the following in order of radiation exposure, where 1 is the lowest radiation exposure dose and 5 is highest radiation exposure dose:

:يرجى ترتيب ما يلي بترتيب التعرض للإشعاع، حيث 1 هي أقل جرعة تعرض للإشعاع و5 هي أعلى جرعة تعرض للإشعاع     

**Table 9:** Question 16

	1	2	3	4	5
Chest CT/الأشعة المقطعية للصدر					
Return flight from Jeddah to Dammam/رحلة جوية من جدة الى دمام					
Chest X-ray/أشعة الصدر					
Annual average dose from radon in the home and workplace in Saudi Arabia/متوسط الجرعة السنوية من غاز الرادون في المنزل ومكان العمل في المملكة العربية السعودة					
Intra-oral dental X-ray/أشعة داخل الفم ،صورة واحدة					

17. If a new dental X-ray device were available that used significantly less radiation, would you be prepared to drive an extra hour to a hospital with this technology?

O Yes / نعم

O No / لا

O Maybe / ربما

18. If a new dental X-ray devise were available that produced better quality imaging, would you be prepared to drive an extra hour to a hospital with this technology?

O Yes / نعم

O No / لا

O Maybe / ربما 

## References

[1] Chen J (2015). Issues and challenges of radiation risk communication to the Public. Radiation Emerg Med.

[2] Hayes SC, Wilson KG, Gifford EV, Follette VM, Strosahl K (1996). Experimental avoidance and behavioral disorders: a functional dimensional approach to diagnosis and treatment. J Consult Clin Psychol.

[3] Dartnell LR (2011). Ionizing radiation and life. Astrobiology.

[4] Charles M (2001). UNSCEAR report 2000: sources and effects of ionizing radiation. United Nations Scientific Comittee on the Effects of Atomic Radiation. J Radiol Prot.

[5] de Gonzalez AB, Darby S (2004). Risk of cancer from diagnostic X-rays: estimates for the UK and 14 other countries. Lancet.

[6] Hall EJ, Brenner DJ (2008). Cancer risks from diagnostic radiology. Br J Radiol.

[7] Balter S (1999). An overview of radiation safety regulatory recommendations and requirements. Cathet Cardiovasc Intervent.

[8] Alyami J, Nassef MH (2022). Assessment of diagnostic radiology facilities technical radiation protection requirements in KSA. Appl Sci.

[9] Shah N, Bansal N, Logani A (2014). Recent advances in imaging technologies in dentistry. World J Radiol.

[10] Shahbazi-Gahrouei D, Gholami M, Setayandeh S (2013). A review on natural background radiation. Adv Biomed Res.

[11] Douple EB, Mabuchi K, Cullings HM (2011). Long-term radiation-related health effects in a unique human population: lessons learned from the atomic bomb survivors of Hiroshima and Nagasaki. Disaster Med Public Health Prep.

[12] Kenny E, Byrne B, Lewis M, King DM (2019). Perception of medical radiation risk in Ireland: results of a public survey. Phys Med.

[13] Bahanan L, Tehsin A, Mousa R, Albadi M, Barayan M, Khan E, Khalifah H (2021). Women's awareness regarding the use of dental imaging during pregnancy. BMC Oral Health.

[14] Kutanzi KR, Lumen A, Koturbash I, Miousse IR (2016). Pediatric exposures to ionizing radiation: carcinogenic considerations. Int J Environ Res Public Health.

